# Maternal Caffeine Consumption during Pregnancy and Risk of Low Birth Weight: A Dose-Response Meta-Analysis of Observational Studies

**DOI:** 10.1371/journal.pone.0132334

**Published:** 2015-07-20

**Authors:** Jongeun Rhee, Rockli Kim, Yongjoo Kim, Melanie Tam, Yizhen Lai, NaNa Keum, Catherine Elizabeth Oldenburg

**Affiliations:** 1 Department of Environmental Health, Harvard T.H. Chan School of Public Health, Boston, Massachusetts, United States of America; 2 Department of Social and Behavioral Sciences, Harvard T.H. Chan School of Public Health, Boston, Massachusetts, United States of America; 3 Department of Global Health and Population, Harvard T.H. Chan School of Public Health, Boston, Massachusetts, United States of America; 4 Department of Health Policy and Management, Harvard T.H. Chan School of Public Health, Boston, Massachusetts, United States of America; 5 Department of Nutrition, Harvard T.H. Chan School of Public Health, Boston, Massachusetts, United States of America; 6 Department of Epidemiology, Harvard T.H. Chan School of Public Health, Boston, Massachusetts, United States of America; East Carolina University, UNITED STATES

## Abstract

Epidemiologic studies have shown inconsistent conclusions about the effect of caffeine intake during pregnancy on the risk of low birth weight (LBW). We performed a meta-analysis and linear-dose response analysis examining the association between caffeine consumption during pregnancy and risk of LBW. PubMed and EMBASE were searched for relevant articles published up to March 2014. Eight cohort and four case-control studies met all inclusion criteria. Using a random-effects model of the twelve studies, the pooled odds ratio (OR) for the risk of LBW comparing the highest versus lowest level of caffeine intake during pregnancy was 1.38 (95% CI: 1.10, 1.73). Linear dose-response analysis showed that every additional 100 mg of caffeine intake (1 cup of coffee or 2 cups of tea) per day during pregnancy was associated with a 3.0% increase in OR for LBW. There was a moderate level of overall heterogeneity with an I-squared value of 55% (95% CI: 13, 76%), and no evidence of publication bias based on Egger’s test (*P* = 0.20) and the funnel plot. Thus, high caffeine intake during pregnancy is associated with a significant increase in the risk of LBW, and this risk appears to increase linearly as caffeine intake increases.

## Introduction

A recommendation to limit caffeine intake during pregnancy was issued by the United States Food and Drug Administration in 1980 [1]. More recently, the American Congress of Obstetricians and Gynecologists reported that moderate caffeine consumption (<200 mg/day) during pregnancy does not seem to be a major risk factor of miscarriage or preterm birth; however it was noted that the association between maternal caffeine intake and infant growth restriction remains undetermined [2].

Pregnant women have slower caffeine metabolism, with 1.5 to 3.5 times longer half-life needed to eliminate caffeine, compared to non-pregnant woman [3]. Caffeine has been detected in the amniotic fluid, umbilical cord, urine, and plasma of fetuses, which suggests that caffeine is easily transmitted across the placenta [4, 5]. The immaturity of a fetus’ liver produces a low level of enzymes necessary for caffeine metabolism, and it leaves neonates at risk of adverse outcomes including low birth weight (LBW) [6]. Infant LBW, defined as a birth weight smaller than 2,500g, is a well-established risk factor associated with several adult diseases, such as hypertension and diabetes mellitus, and obesity [7].

Epidemiologic studies have reported inconsistent conclusions about the effects of caffeine intake during pregnancy on LBW. Larroque et al [8] and Fortier et al [9] reported no association between caffeine consumption and birth weight. However, a meta-analysis of seven studies in 1998 [10] found a significant increase in the risk of LBW associated with caffeine consumption. Recently, Greenwood et al [11] found that consuming an increment of 100 mg/day of caffeine was associated with a 7% increase in the risk of LBW in a dose-response meta-analysis. These publications necessitate a revisit to the risk of maternal caffeine consumption on LBW. Hence, the purpose of our study is to systematically review the literature and perform a meta-analysis, including a dose-response analysis, on maternal caffeine consumption during pregnancy and associated risk of LBW.

## Materials and Methods

### Search strategy

A literature search was performed in PubMed and Embase for all studies published up to March 2014 using the Medical Subject Headings (MeSH) terms or key words *pregnancy*, *pregnancy outcome*, *perinatal*, *maternal*, *caffeine*, *coffee*, *birth weight*, *infant*, and *low birth weight* (S2 File). Five blinded investigators (JR, RK, YK, MT, and YL) independently reviewed the titles, abstracts and full texts using pre-specified eligibility criteria. Additionally, the reference lists of all retrieved articles and previous relevant meta-analysis/review articles were checked to identify additional studies. We followed the guidelines of Meta-analysis Of Observational Studies in Epidemiology (MOOSE) throughout the design, conduct, analysis, and reporting of this meta-analysis [12].

### Study selection

**Fig 1:**
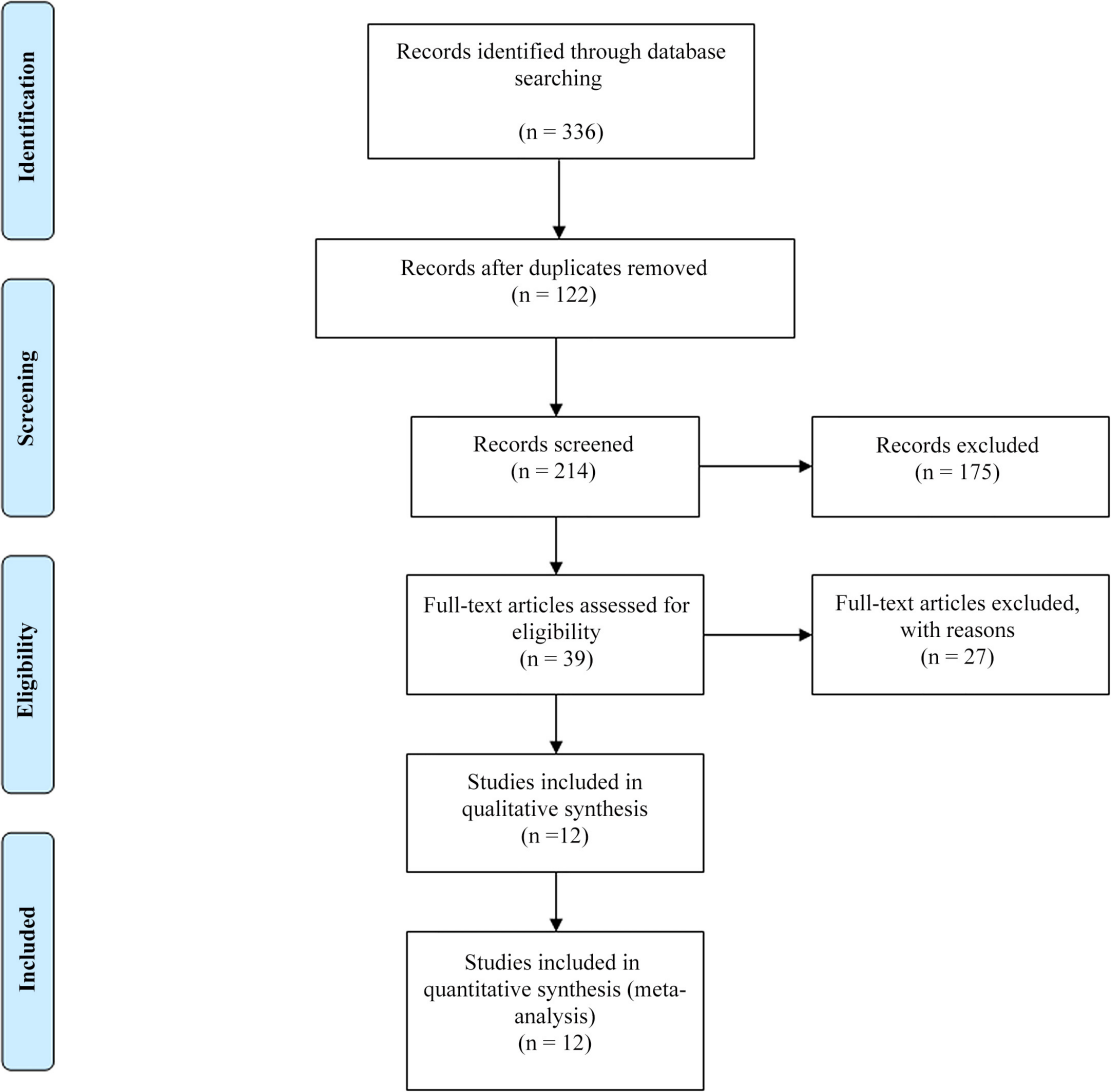
Literature search results for publications related to caffeine consumption during pregnancy and risk of low birth weight.

We included cohort and case-control studies that examined the association between maternal caffeine intake and LBW. All sources of caffeine exposure, such as coffee, tea, cocoa/chocolate and soda drinks, were included. The outcome, LBW, was defined as birth weight smaller than 2,500 grams. We also restricted to studies providing measures of association, relative risk (RR) or odds ratio (OR) estimate and 95% confidence interval (CI), to perform meta-analysis (Fig 1). Studies presenting more than three categories of caffeine consumption, as well as category-specific number of cases and non-cases, the estimates of ORs or RRs, and associated 95% CI, were further eligible to conduct the dose-response meta-analysis. Non-English articles, literature reviews, abstracts, posters, case reports, animal studies, unpublished results, and studies examining birth complications other than LBW (preterm birth, short for gestational age, etc.) were excluded (S1 Table). Study selection was completed through discussion among the five authors.

### Data extraction

From each selected study we extracted the following information: authors, publication year, study design (cohort or case-control), study type (retrospective or prospective), source of caffeine (beverage type), exposure timing (trimester), number of cases and non-cases, sample size, most fully adjusted estimates of association and corresponding 95% CIs, study region, percentage of women age > = 35, measurement of caffeine consumption (validated or not), measurement of outcomes (medical record or not), adjustment for potential confounders (Tables 1 and 2 and S3 Table). For articles that reported cases and non-cases only, we manually calculated the crude effect estimates and 95% CIs [13–15]. For the dose-response meta-analysis, we extracted category-specific doses of caffeine consumption (range, median), the most fully adjusted ORs and their 95% CI (S2 Table). All extracted data were cross-checked at least twice by the authors.

**Table 1:** Cohort studies of caffeine consumption and risk of low birth weight.

Author, publication year, country	N cases, total N	Intake measurement	Trimester considered	Comparisons made by authors	Risk estimate	95% CI	Comparisons made in meta-analysis	Dose-response analysis*	Variables adjusted for
Linn et al, 1982, USA	927, 12205	Coffee, tea †	First	0 cup/d	1		> 400 mg/d vs 0 mg/d		age>35, parity, race, college education, smoking at delivery, alcohol in the first trimester, on welfare, previous stillbirth, previous induced abortion, previous spontaneous abortion, ponderal index
				> 4 cups/d	1.17	0.85, 1.61			
Martin et al, 1987, USA	70, 3654	Coffee, tea, colas, drugs	Throughout pregnancy	0 mg/d	1		> 300 mg/d vs 0 mg/d	√	age, marriage, ethnicity, education, cigarettes, alcohol, marijuana use, parity, previous spontaneous abortion, previous induced abortion, previous stillbirth, weight gain, BMI
				1–150 mg/d	1.4	0.70, 3.00			
				151–300 mg/d	2.3	1.10, 5.20			
				> = 300 mg/d	4.6	2.00, 10.50			
McDonald et al, 1992, Canada	1742, 30,445	Coffee †	Throughout pregnancy	0 cup/d	1		1,000 mg/d vs	√	age, number of prior pregnancies, previous spontaneous abortion, previous LBW infant, pre-pregnancy weight, ethnic group, education, employment at start of pregnancy, smoking, alcohol
				1–2 cups/d	1.05	0.95, 1.16	0 mg/d		
				3–4 cups/d	1.08	0.93, 1.25			
				5–9 cups/d	1.13	0.92, 1.39			
				10 cups/d	1.43	1.02, 2.02			
Eskenazi et al, 1999, USA	307, 7855	Caffeinated coffee, tea, cola	Second	N/A	1		Highest vs Lowest	** **	** **
				N/A	1.17	0.90 to 1.53		** **	** **
Olsen et al, 1991, Denmark	391, 11591	Coffee	First to Second	0–3 cups/d	1		> 800 mg/d vs 150 mg/d	√	smoking, social group, parity, alcohol intake
		(1 cup = 100 mg caffeine)		3–7 cups/d	1.4	1.10, 1.70			
				> 8 cups/d	1.2	0.90, 1.80			
Bracken et al, 2003, USA	108, 2292	Coffee, tea, soda	First and third	0 mg/d	1		> 300 mg/d vs 0 mg/d	√	age, parity, #prior pregnancies, marital status, race, education, height, smoking during third trimester, pre pregnancy weight
				1–149 mg/d	1.45	0.89, 2.35			
				150–299 mg/d	1.59	0.70, 3.60			
				> 300 mg/d	1.32	0.46, 3.78			
Fortier et al, 1993, Canada	321, 6733	Coffee, tea, caffeinated cola, chocolate	Throughout pregnancy	0–10 mg/d	1		> 300 mg/d vs 5 mg/d	√	cigarette consumption, # of previous low birth weight newborns, family income, and parity
				11–150 mg/d	1.27	0.91, 1.76			
				151–300 mg/d	1.25	0.81, 1.93			
				> = 300 mg/d	0.99	0.52, 1.87			** **
Bakker et al, 2010, Netherlands	329, 7083	Coffee or tea (caffeinated and decaffeinated)	Third	< 2 unit/d	1		540 mg/d vs < 180mg/d	√	gestational age at visit, maternal age, educational level, ethnicity, parity, smoking habits, alcohol consumption, height, BMI at intake, nutritional intake, folic acid supplement use, maternal pregnancy complications, and fetal sex.
				2–3.9 unit/d	1.08	0.84 to 1.40			
				4–5.9 unit/d	1.19	0.73 to 1.95			
				> 6 unit/d	2.58	1.26 to 5.30			
Mills et al, 1993, USA	21, 352	Regular or decaffeinated coffee, hot or iced tea, cocoa, regular or cola drinks, other diet drinks, drugs	Throughout pregnancy	0 mg/d					maternal age, income, education, pre-pregnancy weight, height, race, parity, smoking, and alcohol use
				1–99mg/d					
				100–199mg/d					
				200–299mg/d					
				> 300mg/d					

Abbreviations: Confidence interval (CI)

* Check mark (√) indicates studies that are included in the dose-response analysis

† Caffeine consumption was originally reported in cups and was converted as1 cup = 100 mg caffeine

**Table 2:** Case-control studies of caffeine consumption and risk of low birth weight.

Author, publication year, country	N cases, N controls	Intake measurement	Trimester considered	Comparisons made by authors	Risk estimate	95% CI	Comparisons made in meta-analysis	Dose-response analysis*	Variables adjusted for
Caan et al,	130, 135	Caffeinated coffee, tea, cola drinks	First	0 mg/d	1		> 301 mg/d vs 0 mg/d	√	ethnicity, alcohol intake, cigarette use, pregnancy weight, weight gain, parity
				1–300 mg/d	0.9	0.42, 1.92			
				> 301 mg/d	2.94	0.89, 9.65			
Fenster et al,	87, 1143	Caffeinated coffee, tea, soft drinks	First	0 mg/d	1		> 301 mg/d vs 0 mg/d	√	age, parity, race, hypertension during pregnancy, cigarettes smoked, alcohol consumed
				1–150 mg/d	0.78	0.45, 1.35			
				151–300 mg/d	1.07	0.51, 2.21			
				> 301 mg/d	2.05	0.86, 4.88			
Santos et al, 1998, Brazil	394, 787	Caffeinated and decaffeinated coffee, tea, mate, cola soft drinks, drinking chocolate, chocolate, and medicines	Throughout pregnancy	0–99 mg/d	1		> 300 mg/d vs 50mg/d	√	cigarette smoking, pre-gestational weight, skin color, living with partner, place of residence, maternal education, frequency of sexual intercourse in last month of pregnancy
				100–299 mg/d	1.07	0.77, 1.50			
				> 300 mg/d	0.73	0.48, 1.12			
Azzeh et al, 2013, Saudi Arabia	92, 91	Tea ‡	Not specified	< 4 cups/d	1		> 200 mg/d vs = < 200mg/d		twin birth, maternal smoking, fruits intake, milk and dairy product intake, maternal age, weight, height, BMI, family income, education, occupation, diabetes, hypertension, anemia, placental problems, previous LBW, previous pregnancies, mother's age in 1st pregnancy, vegetable intake, meat intake, bread and rice intake, coffee/chocolate/soft drink intake
				> = 4 cups/d	0.99	0.65, 6.19			

Abbreviations: Confidence interval (CI)

* Check mark (√) indicates studies that are included in the dose-response analysis

‡ Caffeine consumption was originally reported in cups and was converted as1 cup = 50 mg caffeine

### Statistical analysis

**Fig 2:**
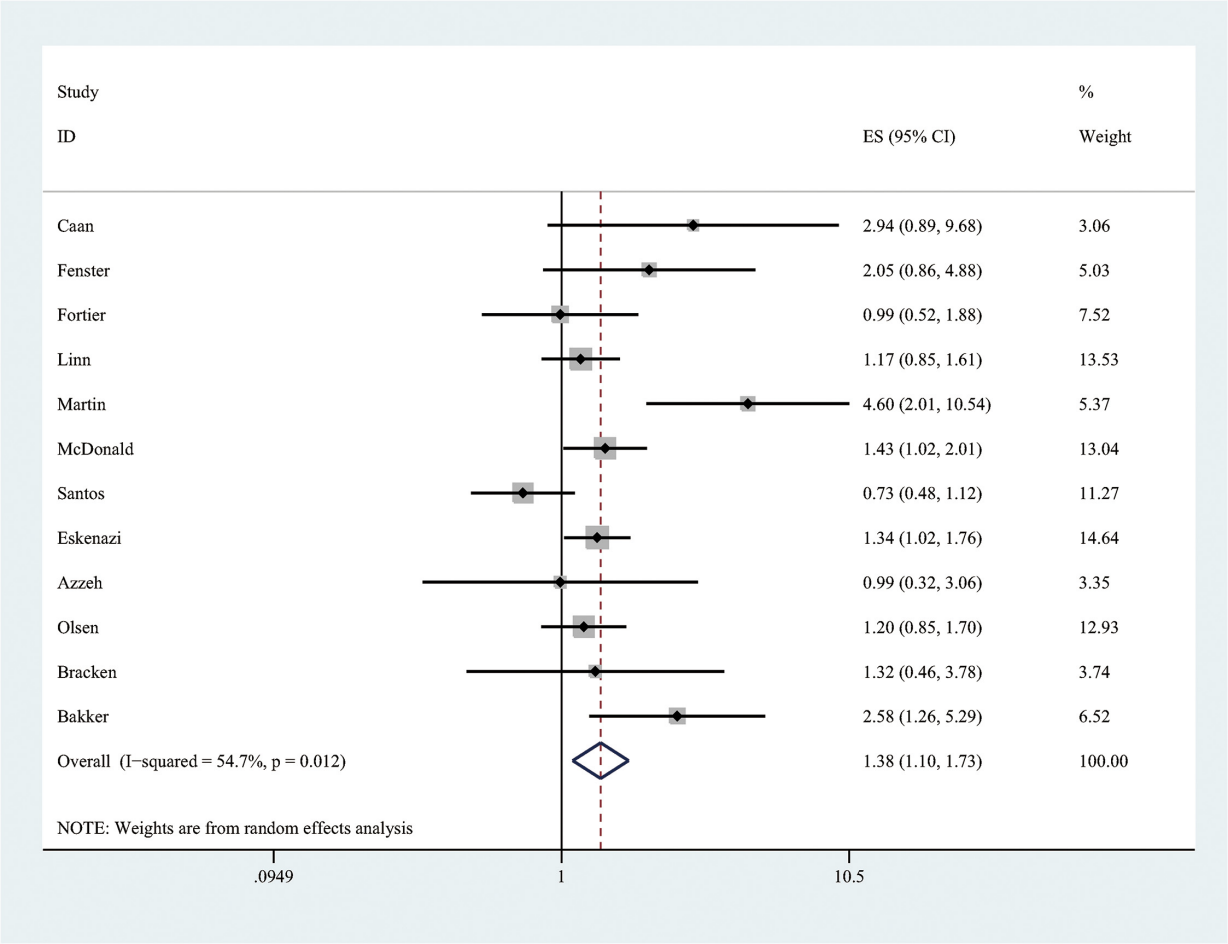
Forest plot of meta-analysis by using random effects model for the effect of maternal caffeine intake during pregnancy on the risk of low birth weight (comparing highest versus lowest levels).

**Fig 3:**
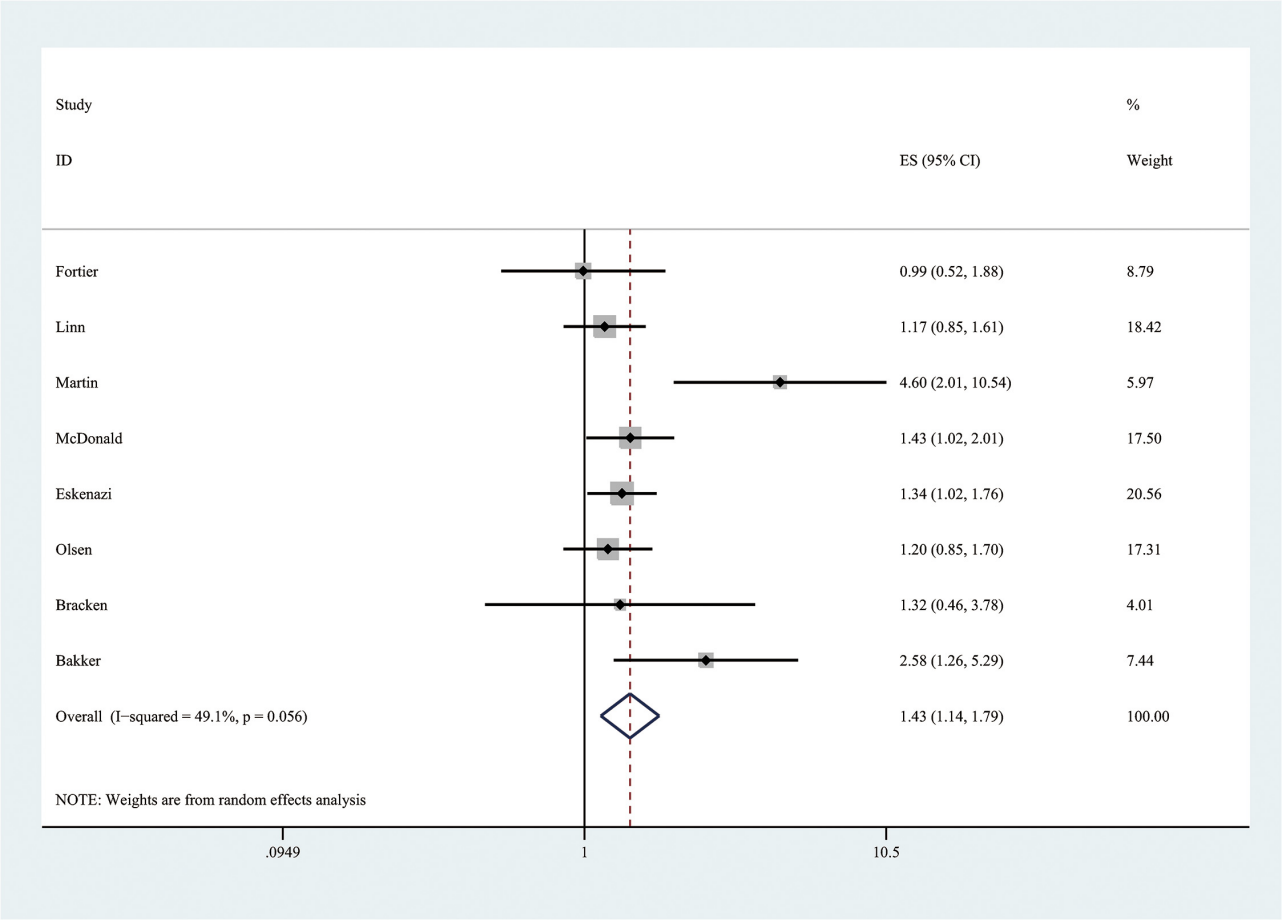
Forest plot of meta-analysis by using random effects model for the effect of maternal caffeine intake during pregnancy on the risk of low birth weight (comparing highest versus lowest levels) among cohort studies.

**Fig 4:**
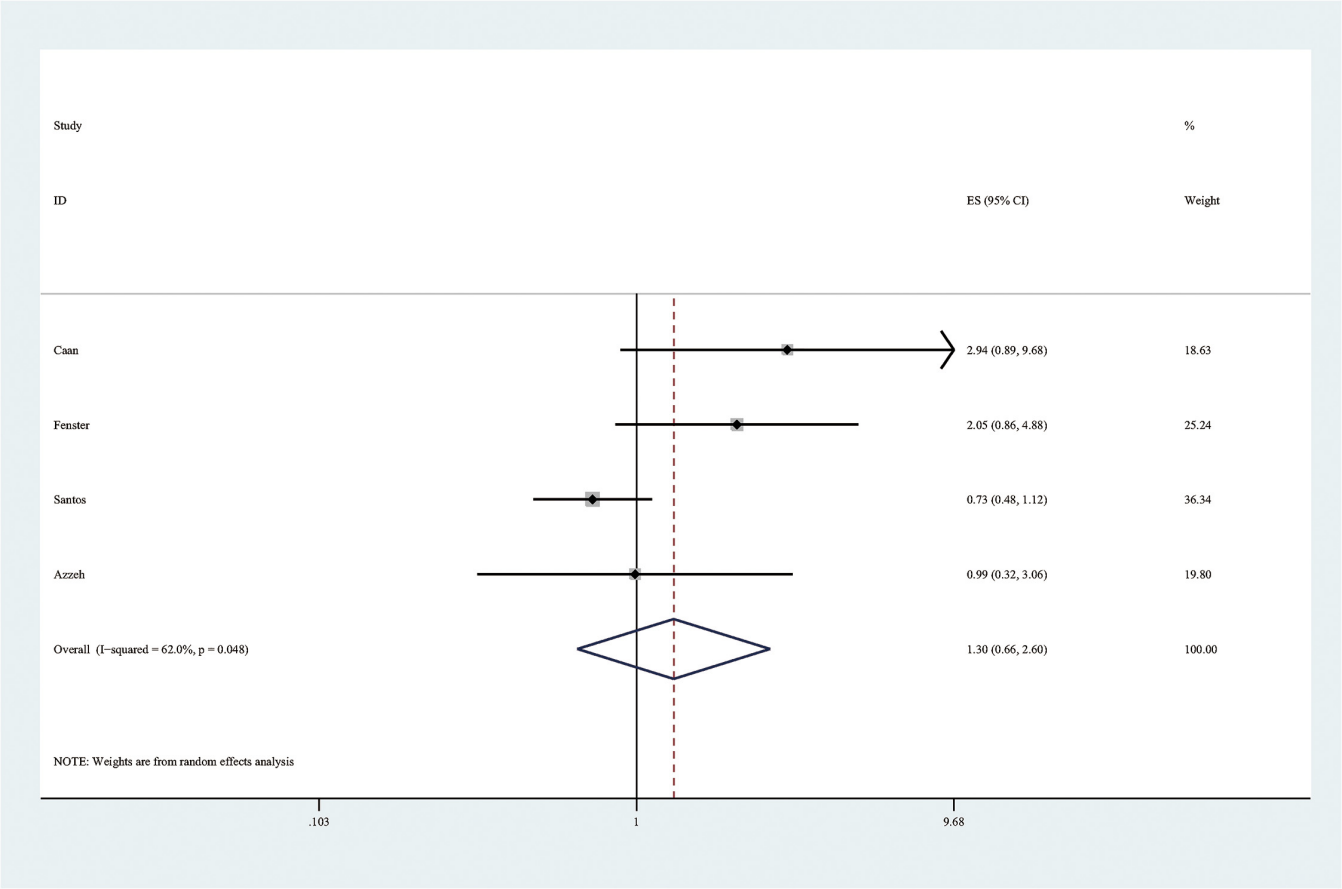
Forest plot of meta-analysis by using random effects model for the effect of maternal caffeine intake during pregnancy on the risk of low birth weight (comparing highest versus lowest levels) among case-control studies.

Firstly, meta-analysis for highest vs lowest exposure was performed. The summary ORs and 95% CIs were calculated by using random effects model, which allows for between-study variation. If consumption was reported as cups/day, we used a conversion equation of 100mg/day of caffeine for 1 cup of coffee and 50mg/day for 1 cup of tea [16]. The forest plot of the overall highest vs lowest meta-analysis is presented (Fig 2 and S3 Table) as well as the plots of subgroup analysis by cohort and case-control study designs (Figs 3 and 4). Every point in the forest plots (Figs 2, 3 and 4) is each study’s odds ratio of LBW comparing highest versus lowest maternal caffeine consumption.

**Fig 5:**
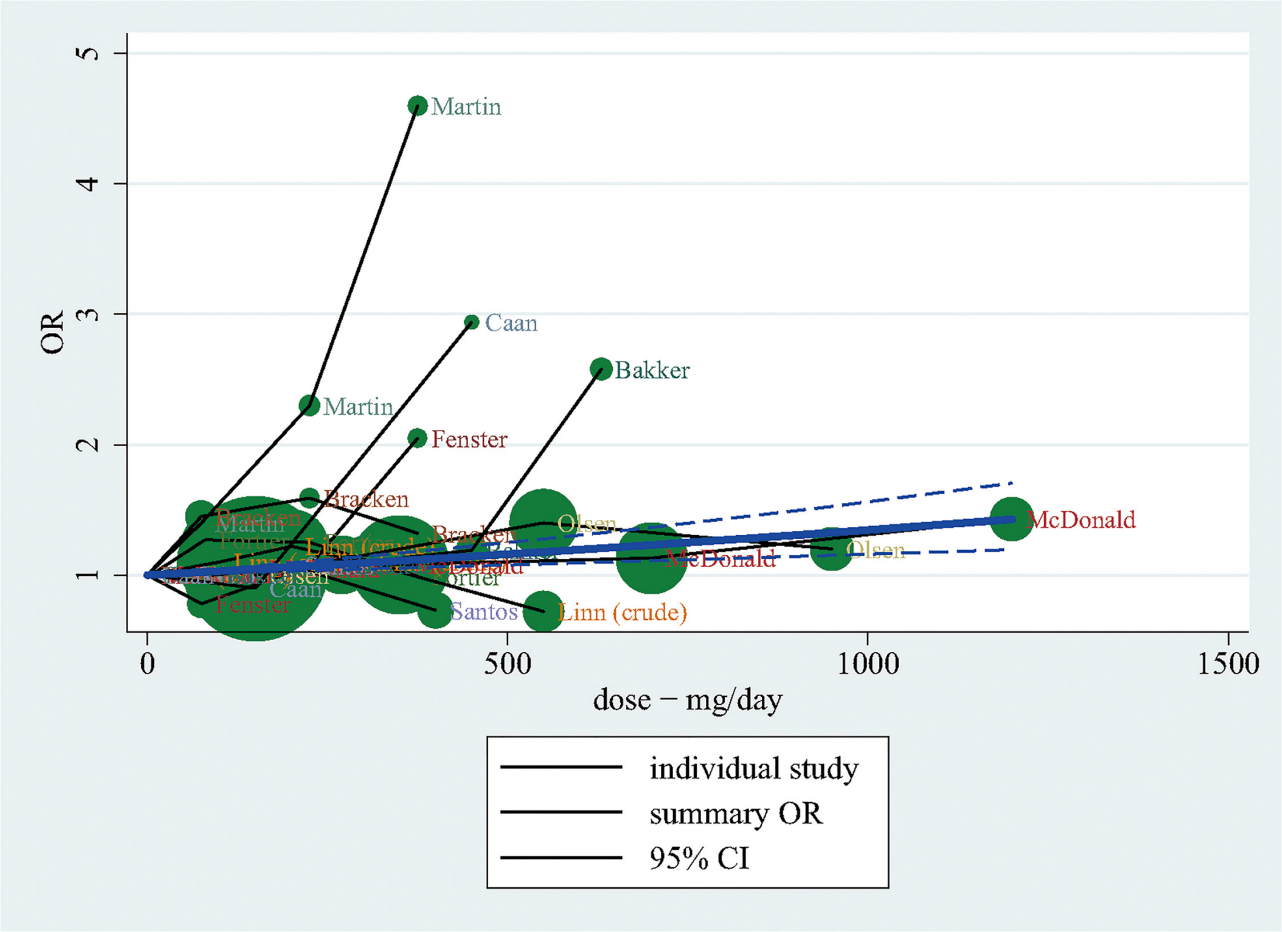
Linear dose-response analysis on the effect of caffeine intake during pregnancy on low birth weight.

A linear dose-response meta-analysis was conducted based on the assumption that there is a linear relationship between maternal caffeine consumption and LBW (Fig 5 and S2 Table). We used the Greenland and Longnecker method [17] to calculate study-specific and overall linear dose-response slopes (ORs) and their 95% CIs. The reference group was the lowest category of caffeine consumption. We calculated the median value of caffeine intakes in each exposure category and matched it to the corresponding OR. For categories with undefined upper boundary (ie. more than 5 cups of coffee per day), we calculated the median value assuming the length of the category to be no different from other defined intervals in the study. We present a spaghetti plot, developed by Ding (E Ding, Harvard School of Public Health, 2014), to summarize the linear dose-response relationship between caffeine consumption and risk of LBW (Fig 5). We used STATA version 13 (StataCorp, College Station, TX) for all statistical analysis.

### Quality assessment

**Fig 6:**
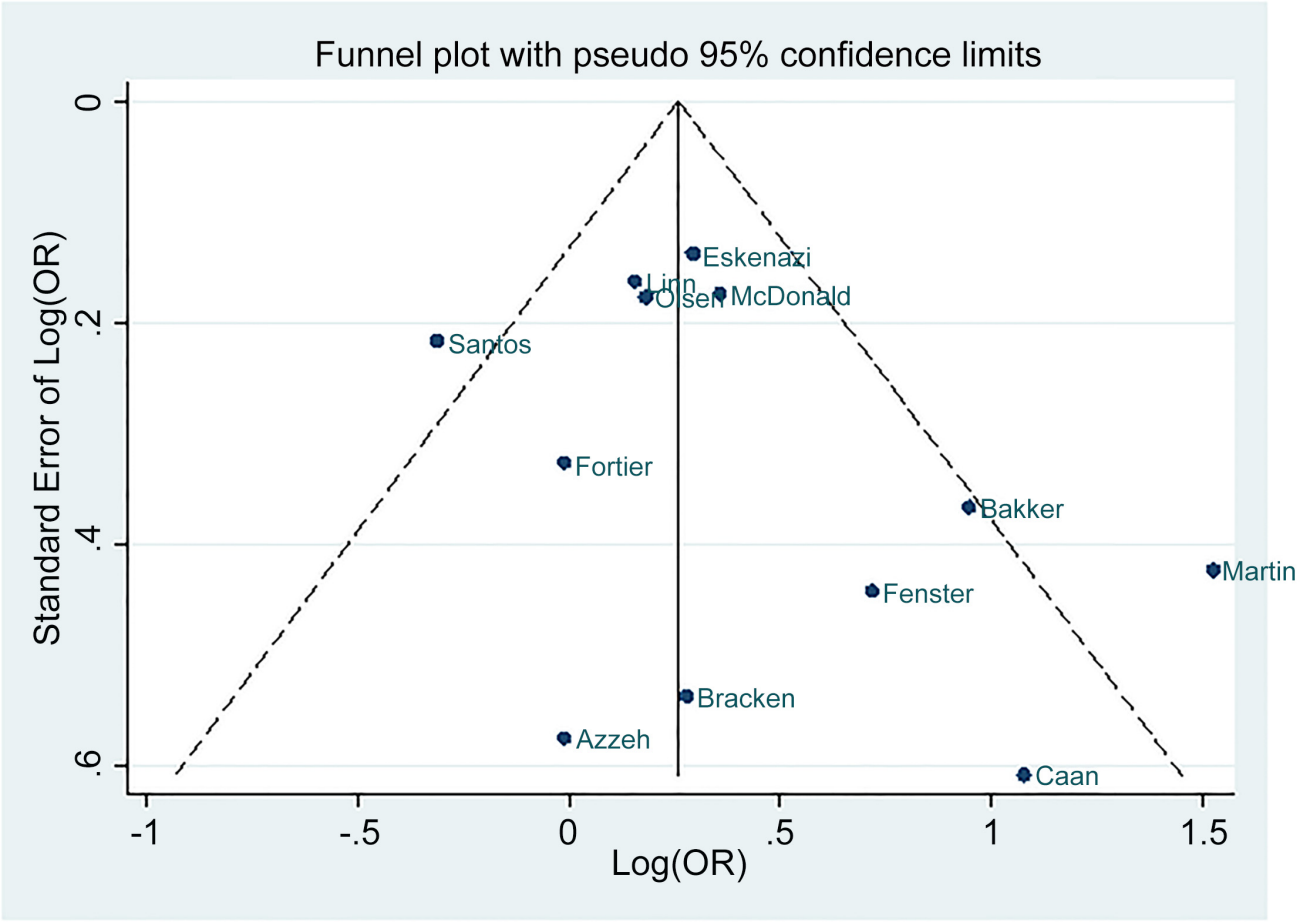
Funnel plot of meta-analysis on the effect of caffeine intake during pregnancy on low birth weight.

Potential publication bias was evaluated by the asymmetrical shape of a funnel plot (Fig 6) and by the p-value from Egger’s test [18]. As sensitivity analysis, we examined the combined risk of LBW excluding studies for which we manually calculated the effect estimates [13–15]. Similarly, we repeated our analysis after omitting studies with very discordant results from the pooled estimates.

Between-study heterogeneity was assessed using Cochran Q-statistic and quantified by Higgins I^2^ statistic and associated 95% CI [19]. According to the Cochrane review standard, a p-value greater than 0.05 for the Q-statistic indicates the absence of heterogeneity and an I^2^ statistic of less than 60% represents moderate or no heterogeneity. Sources of heterogeneity were explored by performing meta-regression and subgroup analyses with respect to study design (case-control, cohort), study region (North America, other regions), exposure measurement assessment (validated or not), sources of caffeine (coffee only, tea only, all types), proportion of women aged 35 (<10%, = 10%), exposure timing (first trimester, across the entire pregnancy or other trimesters), publication year (1980s, 1990s, 2000s), and the level of confounder adjustment (smoking, maternal weight, alcohol consumption, SES, race/ethnicity) (Table 3).

**Table 3:** Subgroup meta-analyses of maternal caffeine intake during pregnancy and LBW.

Study characteristics	# of Studies	N of Cases	Total N	Pooled OR	95% CI	*P* for Heterogeneity	I^2^	95% CI
			Study design (*P* for interaction = 0.54)					
Cohort	8	3,887	74,885	1.43	1.14, 1.79	0.06	49%	0, 77%
Case-control	4	703	2,156	1.3	0.66, 2.60	0.05	62.00%	0, 87%
			Region (*P* for interaction = 0.37)					
North America (US, Canada)	8	3,713	63,402	1.48	1.15, 1.91	0.08	44%	0, 75%
Other regions	4	877	16,086	1.19	0.72, 1.95	0.03	67.70%	6, 89%
			Aged> = 35 composition (*P* for interaction = 0.84)					
<10%	6	893	23,816	1.52	1.04, 2.22	0.03	58.60%	0, 83%
>10%	4	3,303	53,280	1.32	1.08, 1.62	0.73	0%	0, 85%
			Exposure validation (*P* for interaction = 0.86)					
validated	3	516	6,684	1.29	0.81, 2.04	0.42	0%	0, 90%
not validated	9	4,074	72,804	1.4	1.08, 1.83	0	64.50%	27, 83%
			Sources of caffeine (*P* for interaction = 0.55)					
All	9	2,365	35,349	1.49	1.07, 2.06	0	66.10%	31, 83%
Coffee only	2	2,133	42,036	1.31	1.03, 1.67	0.48	-	
Tea only	1	92	2,103	0.99	0.32, 3.06	-	-	
			Timing of the exposure (*P* for interaction = 0.70)					
First trimester	3	3,338	60,785	1.55	0.92, 2.62	0.2	38.80%	0, 81%
All or other trimesters	8	1,252	18,703	1.38	1.03, 1.85	0	66.30%	29, 84%
			Publication Year (*P* for interaction = 0.78)					
1980s	2	997	15,859	2.19	0.58, 8.36	0.003	89%	-
1990s	7	3,393	59,234	1.23	0.97, 1.55	0.1	44.50%	0, 77%
2000s	3	200	4,395	1.72	0.96, 3.09	0.31	15.80%	0, 91%
			Adjustment for alcohol drinking (*P* for interaction = 0.08)					
Yes	7	3,368	62,532	1.7	1.24, 2.34	0.02	59.40%	7, 82%
No	5	1,222	16,956	1.05	0.78, 1.42	0.21	32.10%	0, 74%
			Adjustment for smoking (*P* for interaction = 0.88)					
Yes	11	307	7,855	1.41	1.07, 1.85	0.01	58.70%	20, 79%
No	1	4,283	71,633	1.34	1.02, 1.76	-	-	
			Adjustment for maternal weight (*P* for interaction = 0.39)					
Yes	5	2,487	39,791	1.19	0.76, 1.85	0.08	52.90%	0, 83%
No	7	2,103	39,697	1.5	1.13, 2.00	0.02	58.70%	5, 82%
			Adjustment for Ethnicity (*P* for interaction = 0.32)					
Yes	8	3,479	55,625	1.62	1.11, 2.36	0	69.60%	37, 85%
No	4	1,111	23,863	1.24	1.02, 1.52	0.81	0.00%	0, 85%
			Adjustment for SES (*P* for interaction = 0.52)					
Yes	7	3,602	61,381	1.28	1.08, 1.53	0.5	0.00%	0, 71%
No	5	988	18,107	1.71	0.96, 3.05	0.3	78.90%	50, 91%

Abbreviations: Confidence interval (CI)

## Results

### Literature search and study selection

The searching scheme resulted in a total of 336 studies. A total of 13 studies, nine cohort [6, 9, 13–16, 20–22] and four case-control studies [23–26], were included with a total of 4,919cases (Fig 1). Of these, three cohort [9, 13, 15] and one case-control [26] studies had not been incorporated in the recent meta-analysis [11]. Mill et al. [15] was not included for meta-analysis since it did not have desired outcomes as defined by our inclusion criteria. For dose-response analysis, ten studies, seven cohort [6, 9, 14, 16, 20–22] and three case-control studies [23–25] with a total of 4,499 cases, met the eligibility criteria.

### Overall effect estimate (highest versus lowest) & dose-response analysis

The pooled estimate of the twelve studies presented that maternal caffeine consumption during pregnancy was associated with increased risk of LBW (pooled OR: 1.38, 95% CI: 1.10, 1.73). The linear dose-response analysis showed that one additional cup of coffee or two additional cups of tea per day during pregnancy was associated with a 3.0% increase in the OR for LBW (pooled OR: 1.03, 95% CI: 1.01, 1.05).

### Test of heterogeneity and subgroup analysis

The test of heterogeneity resulted in a moderate level of heterogeneity (*P* = 0.01, I^2^ = 55%, 95% CI: 13, 76%). The meta-regression tests showed that none of the study characteristics, including study design, proportion of women aged 35 and older, region, exposure assessment, sources of caffeine, timing of the exposure, publication year, and confounders adjustment status, significantly modified the pooled estimate for the effect of maternal caffeine intake on the risk of LBW. The results of the meta-regression tests for heterogeneity and subgroup meta-analysis are displayed in Table 3.

### Influence test & publication bias

The sensitivity analysis showed that there were no changes in directionality and significance of the pooled ORs of high vs low meta-analysis after excluding studies with manually calculated effect estimate (Eskenazi et al. [13] (pooled OR = 1.41, 95% CI: 1.07, 1.85) and Linn et al. [14] (pooled OR = 1.43, 95% CI: 1.10, 1.87)), and most influential study (Martin et al. [20] (pooled OR = 1.27, 95% CI: 1.05, 1.52). Similarly, removing Linn et al. [14] did not change the directionality and significance of the OR for the linear dose-response meta-analysis (OR = 1.03 95% CI: 1.02, 1.05). However, after removing Martin et al. [20], the OR was no longer significant (OR = 1.07 95% CI = 0.18, 6.19).

Visual inspection of the funnel plot suggested that the studies were nearly symmetrically distributed around the log of the pooled estimate, and Egger’s tests formally presented that there was no significant evidence of publication bias (*P* = 0.20).

## Discussion

This meta-analysis of twelve studies identified an overall 37.8% increase (Fig 2) in the odds of LBW among women in the highest caffeine intake group compared to those in the lowest group. A dose-response analysis based on ten studies found a 3.0% increase (Fig 5) in the odds of LBW for every 100 mg of caffeine consumed per day during pregnancy, which is equivalent to about one cup of coffee or two cups of tea. The effect size of our high vs low meta-analysis is relatively small compared to well recognized risk factors of LBW, such as active maternal smoking [27–30]. Jaddoe et al. [27] and Horta et al. [28] found active maternal smoking during pregnancy increased the risk of LBW incidence by 75% and 59%, respectively. However, the OR of LBW among pregnant women exposed to environmental tobacco smoke (ETS) is similar to our result [29, 31, 32]. Salmasi et al. [31] and Leonardi-Bee [32] reported ETS exposure increased the risk of LBW births by 16% and 22%, respectively. Although the effect size is small in our dose-response analysis, the result is more precisely estimated (Fig 5), compared to conventional analysis (Fig 2), by using pooling data from ten studies and indicated that there is a significant association even with very small dose of exposure. Since there is no evidence of threshold effect or plateau in the linear dose-response curve (Fig 5), recommendations to pregnant women regarding caffeine intake should consider the absolute risk of increasing maternal caffeine consumption.

Our findings are in agreement with a meta-analysis conducted by Fernandes et al. [10] in 1998, which reported an increased risk of LBW among pregnant women who consumed more than 150 mg of caffeine per day. Also, Sengpiel et al. [33] reported a 21–28 g decrease in birth weight for each additional 100 mg of caffeine consumed per day. Similar results have been reported in several recent studies [34, 35], while others have not found a significant association to exist [36, 37].

The effect of caffeine consumption during pregnancy is of public health concern because caffeine passes through placental barriers [38]. The cytochrome P450 1A2 enzyme (CYP1A2) predominantly metabolizes caffeine [39]. Tsutsumi et al. [40] reported that CYP1A2 activity in early (8–16 weeks) and late (32–39 weeks) pregnancy is reduced by 35% and 52%, respectively. During pregnancy, the half-life of caffeine increases, which causes caffeine to be retained in the body longer [41, 42]. Caffeine can then cross the placenta and be present in the plasma of newborns [5]. Since the levels of CYP1A2 are believed to be low in the placenta and fetus [43], the fetus can be exposed to caffeine for a long period of time. The pharmacological effects of caffeine related to fetal growth are the blockade of adenosine receptors and the inhibition of cyclic nucleotide phosphodiesterase (PDE) [44]. When caffeine acts as an antagonist of adenosine receptor, adenosine is unable to regulate the local blood flow during hypoxia [9]. The acute maternal hypoxia can negatively impact the fetal cardiovascular function and fetal growth [45]. Also, when PDE is inhibited by caffeine, the levels of cyclic adenosine monophosphate (cAMP) will be increased because PDE degrades cAMP, which may interfere with fetal growth. For example, Bistoletti et al [46] explained that fetal asphyxia is associated with higher cyclic AMP levels.

Of the studies included in this meta-analysis, four [6, 13, 20, 21] reported a significant positive association between caffeine intake and LBW while eight found no significant association [9, 14, 16, 22–26]. In case-control studies, mothers with an LBW outcome are more likely to report their caffeine consumption to be less than true amount, which may attenuate the effect estimate towards the null or bias it towards inverse association [47]. Attenuated linear dose-response line was shown (Fig 5) rather than strong linear line when mothers who consumed high levels of caffeine under-reported their actual consumption. This could explain why we observed different results for case-control and cohort studies in our high vs low meta-analysis. Among the cohort studies, there was a significant increase in risk of LBW among mothers who consumed higher amounts of caffeine. Among the case-control studies, the effect was no longer significant, although the directionality of the association was consistent.

Overall, there was a moderate level of heterogeneity between the studies, but we were not able to identify significant sources of heterogeneity after performing meta-regression tests based on different study characteristics. However, removing the most influential study, Martin et al [20], reduced the level of heterogeneity from I-squared of 55% to 34%. After reviewing its study design and adjusted confounders, we were not able to identify characteristics of this study [20] that may have caused it to be particularly influential. Removing another study that did not provide an adjusted OR, Eskenazi et al. [13] Linn et al. [14], did not change the directionality or significance of the results.

This meta-analysis also considered possible confounders of the relationship between caffeine intake and LBW by using adjusted ORs reported by the studies (Tables 1 and 2). The most common confounders adjusted for were smoking status, parity, alcohol use, and ethnicity. Maternal age, weight, and trimester were also adjusted for in many studies. Levels of smoking, alcohol use, and maternal age have previously been found to have a positive correlation with levels of caffeine consumption [48].

This meta-analysis has a few limitations. Frequency, quantity, and sources of caffeine intake during pregnancy were self-reported by the mothers or expectant mothers in all accepted studies. Thus, recall bias might have occurred in terms of exposure assessment, especially for the seven retrospective studies [9, 14, 21, 23–26]. Most of the studies did not conduct exposure measurement validation. However, Fenster et al. [24] and Bracken et al. [16] reported that retrospective data was reliable. Fenster et al. [24] found that 77% of the respondents could reproduce their caffeine consumption record from 6 months earlier within one cup of coffee. Bracken et al. [16] observed that prospective data collected at 28 and 36 weeks of pregnancy were similar to respective data gathered retrospectively. It is not possible to perform individual exposure quality assessment, but misclassification of exposure would have likely occurred randomly, causing non-differential bias. Exposure validation was not found to be a source of heterogeneity in our pooled estimates.

Another limitation is that studies differed in the units used to measure caffeine intake. The amount of caffeine consumption was measured in cups per day by four studies [14, 21, 22, 26], while the other studies reported milligrams per day. To make these estimates comparable for dose-response analysis, we used conversion factors of 100mg/day for 1 cup of coffee and 50mg/day for 1 cup of tea. Since each study had different conversion factors, exposure levels may have been slightly under- or overestimated. For example, Fenster et al. [24] assumed a caffeine content of 107mg/cup of coffee and 34mg/cup of tea, and Bakker et al. [6] estimated that 1 cup of caffeinated coffee contains 90mg caffeine However, we believe that an approximate difference of 10mg for estimating one cup of coffee or tea would not lead to clinically relevant differences in the results.

A strength of this meta-analysis is the extensiveness of the literature search; all studies ever conducted prior to March 2014 were considered for inclusion. Our findings not only reaffirm the recent meta-analysis results [11, 49] that identified an inverse relationship between caffeine consumption and birth weight, but also induce a comprehensive conclusion by including more studies. The quality of outcome measurement in all studies was reliable as they were collected by birth certificate, from hospitals, or were confirmed by physicians. Furthermore, evaluation of the funnel plot and Egger’s test found no evidence of publication bias.

The results of our meta-analysis suggest that consuming 100 mg of caffeine per day may lead to a small, but significant, increase in the risk of LBW. It is important that women are aware of the effects that caffeine may have on their infants’ health and make appropriate adjustments to their levels of consumption.

## Supporting Information

S1 File PRISMA 2009 Checklist. (DOC)

S2 File Medical Subject Heading (MeSH) terms and title/abstract (tiab) keywords in PubMed and Embase search strategy. (DOCX)

S1 Table The reasons of excluding 27 articles. (TIF)

S2 Table Data used for dose-response analysis. (TIF)

S3 Table Data used for meta-analysis (highest vs lowest). (TIF)
