# Supplementary material for: Maternal Caffeine Consumption during Pregnancy and Risk of Low Birth Weight: A Dose-Response Meta-Analysis of Observational Studies
Source: PLoS One. 2015 Jul 20;10(7):e0132334. doi: 10.1371/journal.pone.0132334 (PMC4507998; doi:10.1371/journal.pone.0132334)
Supplement: S2 File — (DOCX) [file pone.0132334.s002.docx]

S2.

| Medical Subject Heading (MeSH) terms and title/abstract (tiab) keywords in PubMed | | |
| --- | --- | --- |
|  | ("Pregnancy"[Mesh:NoExp] OR "Pregnancy Outcome"[Mesh] OR pregnancy[tiab] OR pregnant[tiab] OR perinatal[tiab] OR maternal[tiab]) AND ("Caffeine"[Mesh] OR caffeine[tiab] OR coffee[ti] OR coffee[Mesh]) AND ("Birth Weight"[Mesh] OR "Infant, Low Birth Weight"[Mesh] OR birth weight[tiab]) NOT ("Animals"[Mesh] NOT "Humans"[Mesh]) | |
| Embase search strategy | | |
|  | | ('pregnancy'/exp OR 'pregnancy outcome'/de OR 'maternal nutrition'/de AND [embase]/lim) AND ('caffeine'/de OR 'coffee'/de AND [embase]/lim) AND ('birth weight'/exp AND [embase]/lim) |
