# Supplementary figures and images for: Maternal Caffeine Consumption during Pregnancy and Risk of Low Birth Weight: A Dose-Response Meta-Analysis of Observational Studies
Source: PLoS One. 2015 Jul 20;10(7):e0132334. doi: 10.1371/journal.pone.0132334 (PMC4507998; doi:10.1371/journal.pone.0132334)

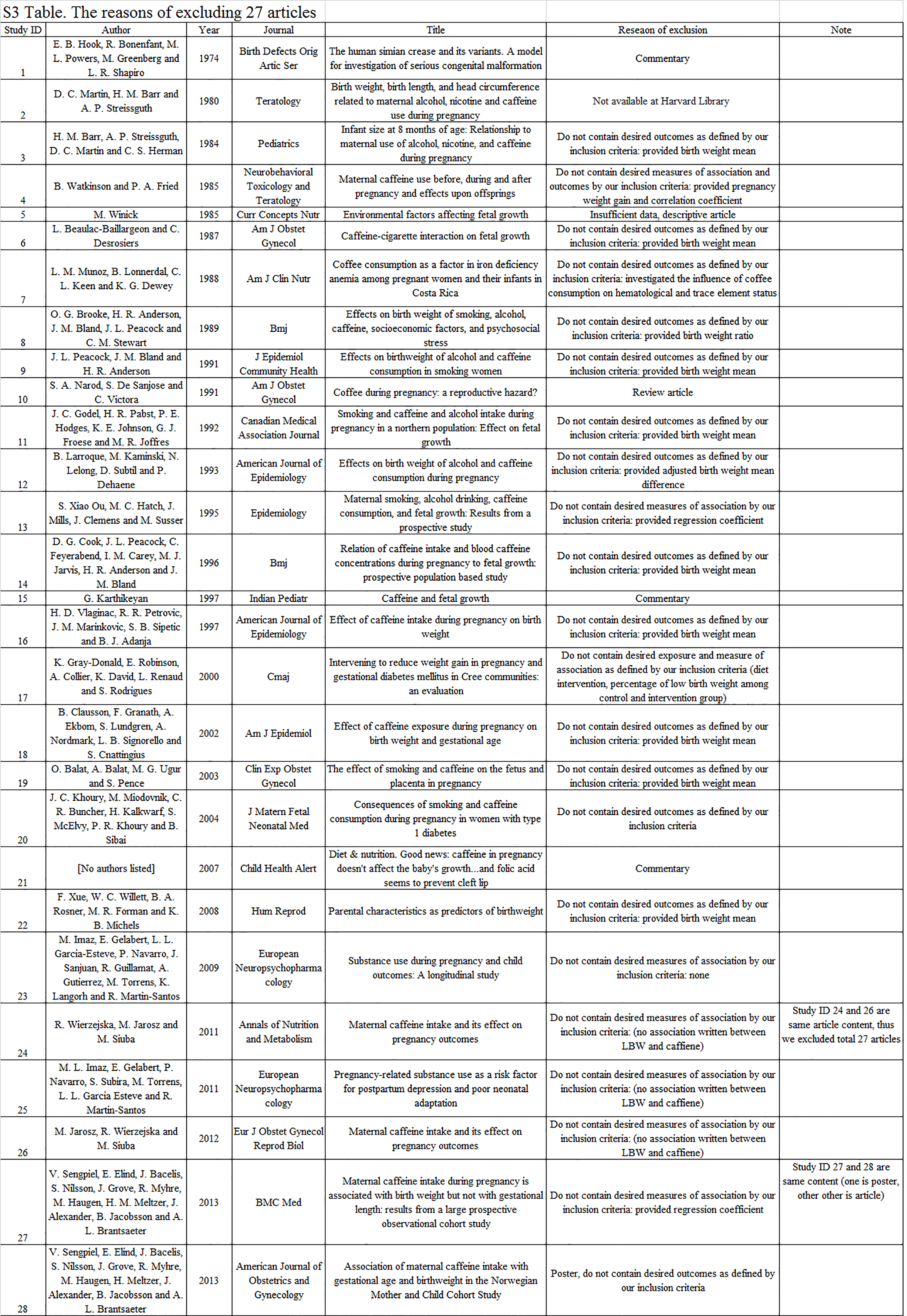

Supplement: S1 Table — (TIF) [file pone.0132334.s003.tif]

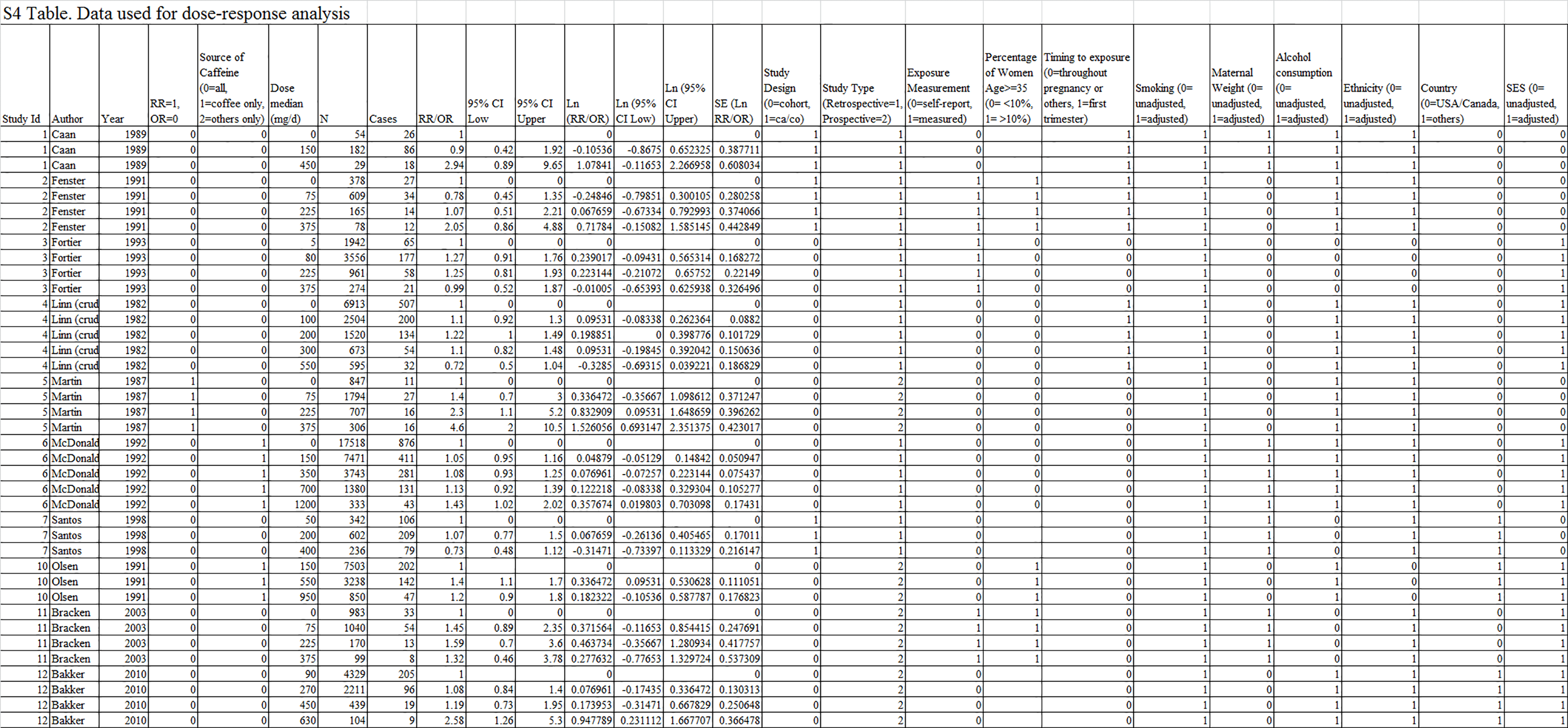

Supplement: S2 Table — (TIF) [file pone.0132334.s004.tif]

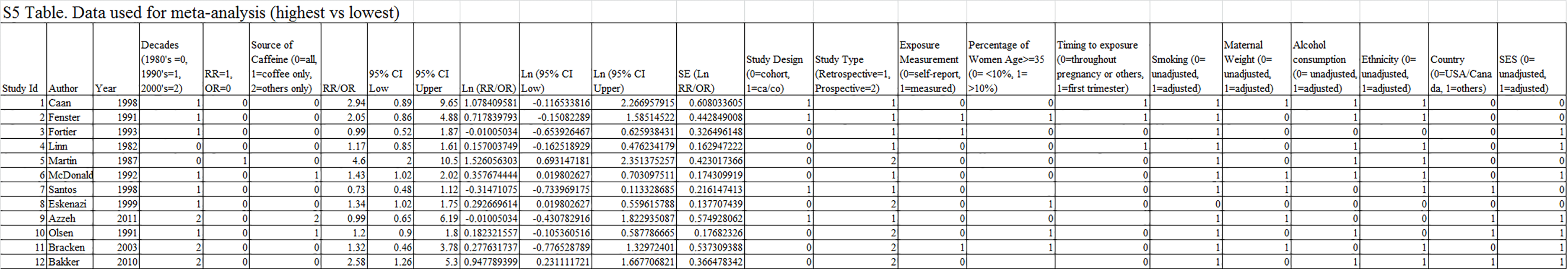

Supplement: S3 Table — (TIF) [file pone.0132334.s005.tif]
